# Investigating a Weakly Informative Prior for Item Scale Hyperparameters in Hierarchical 3PNO IRT Models

**DOI:** 10.3389/fpsyg.2017.00123

**Published:** 2017-02-06

**Authors:** Yanyan Sheng

**Affiliations:** Department of Counseling, Quantitative Methods and Special Education, Southern Illinois UniversityCarbondale, IL, USA

**Keywords:** item response theory, Gibbs sampling, three-parameter models, hyperprior, scale hyperparameter, half-*t*, half-Cauchy, half-normal

## Abstract

The half-*t* family has been suggested for the scale hyperparameter in Bayesian hierarchical modeling. Two parameters define a half-*t* distribution: the scale *s* and the degree-of-freedom ν. When *s* is set at a finite value that is slightly larger than the actual standard deviation of the parameters, the half-*t* prior density can be vaguely informative. This paper focused on such densities, and applied them to the hierarchical three-parameter item response theory (IRT) model. Monte Carlo simulations were carried out to investigate the performance of such specifications in parameter recovery and model comparisons under situations where the actual variability of item parameters varied, and results suggest that the half-*t* family does offer advantages over the commonly adopted uniform or inverse-gamma prior density by allowing the variability for item parameters to be either very small or large. A real data example is also provided to further illustrate this.

## 1. Introduction

With current enhanced computational technology and the emergence of Markov chain Monte Carlo (MCMC) simulation techniques (e.g., Chib and Greenberg, 1995), the methodology for parameter estimation with item response theory (IRT) models has rapidly moved to a fully Bayesian approach. One of the many advantages that this approach offers in the simultaneous estimation of both item and person parameters is the flexibility of setting prior distributions for model parameters or hyperparameters. The existing literature in Bayesian statistics (Gelman et al., 2003) offers two general options in specifying prior distributions by choosing between fully informative priors using application-specific information and non-informative priors. Each of these is adopted depending on the availability of prior information. However, when prior information is desired but not readily available, neither would provide a common solution for various actual situations. The problem with the former, in particular, is that misspecification tends to result in biased estimates and hence incorrect inferences (e.g., Mislevy, 1986). This paper focuses on something in the middle, namely, a somewhat informative prior distribution that can be used in a wide range of applications.

The fully Bayesian estimation procedure has been developed for the three-parameter normal ogive (3PNO; Lord, 1980) model by Sahu (2002; see also Johnson and Albert, 1999) generalizing the approach for the two-parameter model by Albert (1992). The procedure has been further implemented in some applications, e.g., Béguin and Glas (2001) and Glas and Meijer (2003). However, this specification where the hyperparameters take specific values causes problems when prior distributions for the item slope and intercept parameters are not strongly informative. Specifically, studies have shown that improper non-informative prior densities for the item slope and intercept parameters result in an undefined posterior distribution, which gives rise to unstable parameter estimates (Sheng, 2008, 2010). Even with proper non-informative prior densities, the procedure either fails to converge or requires an enormous number of iterations for the Markov chain to reach convergence (Sheng, 2010). Sheng (2013) shows that if one specifies prior distributions for the hyperparameters of the item parameters, instead of setting values for them, the problem can be resolved. This type of hierarchical modeling allows a more objective approach to inference by estimating the parameters of prior distributions from data rather than specifying them using subjective information.

Research on the effect of prior distributions for hyperparameters in Bayesian hierarchical models advises caution in choosing a prior distribution for the scale hyperparameter, as certain specifications of the hyperpriors may cause problems in inference (Brown and Draper, 2006; Gelman, 2006). In the Bayesian literature and software, various non-informative prior distributions have been suggested for the variance parameter in hierarchical linear models, including an improper uniform prior on σ (Gelman et al., 2003), an improper uniform prior on log(σ), and a conjugate inverse-gamma (0.001, 0.001) prior (Spiegelhalter et al., 1994, 2003). Gelman (2006), however, in an attempt to illustrate the performance of these prior distributions for σ near zero, which is where classical and Bayesian inferences differ the most (see Brown and Draper, 2006), pointed out problems with the latter two, especially with the inverse-gamma family of non-informative prior distributions. As Gelman (2006) stated, the problem with the uniform prior distribution on log(σ) is that it results in an improper posterior distribution (p. 521), and the problem with the inverse-gamma family is that inferences are sensitive to the choice of the hyperparameters when low values of σ are possible (p. 522). With respect to the proper non-informative prior, he recommended the use of a half-*t* family, which is inherently conjugate (see Gelman, 2006, for a detailed illustration) and is preferred over other parametric family for the hyperprior distributions because flat-tailed distributions allow for robust inference (Berger and Berliner, 1986). When the scale of the half-*t* distribution is set to a finite value that is slightly larger than the actual variability of the parameters, the resulting prior density can be vaguely informative.

In view of the above, the purpose of this study is to develop Bayesian hierarchical 3PNO IRT models with such half-*t* densities being the item scale hyperpriors and further investigate their performance in estimating model parameters as well as in providing model-data fit under different test situations where the actual variability of item parameter varies.

The remainder of the paper is organized as follows. Section 2 describes the hierarchical 3PNO IRT model and the Gibbs sampling procedure where half-*t* hyperpriors are assumed for the scale parameters for item slopes and intercepts. Then, two simulation studies were carried out to evaluate the performance of this model specification in parameter recovery as well as to compare it with other model specifications with uniform or inverse-gamma prior densities. The methodology and results of these simulation studies are presented in Sections 3 and 4, respectively. Section 5 gives an example where the model specification under investigation is implemented on a subset of *College Basic Academic Subjects Examination* (*CBASE*; Osterlind, 1997) *English* data. Finally, a few summary remarks are provided in Section 6.

## 2. Model and the Gibbs sampling procedure

Before the study is further described, the hierarchical 3PNO model is briefly illustrated. Suppose a test consists of *k* binary response items (e.g., multiple-choice items), each measuring a single unified latent trait, θ. Let ***y*** = [*y*_*ij*_]_*n* × *k*_ denote a matrix of *n* responses to the *k* items where *y*_*ij*_ = 1 (*y*_*ij*_ = 0) if the *i*-th person answers the *j*-th item correctly (incorrectly) for *i* = 1, …, *n* and *j* = 1, …, *k*.

The probability of person *i* obtaining a correct response to item *j* can be defined as

(1) $$\begin{array}{l} P\left(y_{ij}=1|\theta_{i},\alpha_{j},\beta_{j},\gamma_{j}\right)=\gamma_{j}+\left(1-\gamma_{j}\right)\Phi \left(\alpha_{j}\theta_{i}-\beta_{j}\right),\;0\leq \gamma_{j}<1 \end{array}$$

for the 3PNO IRT model, where Φ denotes the normal CDF, θ_*i*_ is a scalar latent trait parameter, α_*j*_ is a scalar slope parameter describing the item discrimination, β_*j*_ is the intercept parameter associated with item difficulty, and γ_*j*_ is a pseudo-chance-level parameter indicating that the probability of correct response is < zero even for those with very low trait levels. This model is applicable for objective items, such as multiple-choice or true-or-false items where an item is too difficult for some examinees.

To implement Gibbs sampling to the 3PNO model defined in (1), two latent variables, *Z* and *W*, are introduced such that *Z*_*ij*_ ~ *N*(η_*ij*_, 1) (Albert, 1992; Tanner and Wong, 1987), where η_*ij*_ = α_*j*_θ_*i*_ − β_*j*_, and *W*_*ij*_ = 1 (*W*_*ij*_ = 0) if person *i* knows (does not know) the correct answer to item *j* with a probability density function

(2) $$\begin{array}{l} P\left(W_{ij}=w_{ij}|\eta_{ij}\right)=\Phi {\left(\eta_{ij}\right)}^{w_{ij}}{\left(1-\Phi \left(\eta_{ij}\right)\right)}^{1-w_{ij}}. \end{array}$$

Prior densities *p*(**θ**), *p*(**ξ**) and *p*(γ) can be assumed for θ_*i*_, **ξ**_*j*_ and γ_*j*_, respectively, where $\xi_{j}=\left(\alpha_{j},\beta_{j}\right)\prime$. Here we focus on the normal conjugate priors for **ξ**_*j*_ so that $\alpha_{j}\sim N_{\left(0,\infty \right)}\left(\mu_{\alpha },\sigma_{\alpha }^{2}\right)$, $\beta_{j}\sim N\left(\mu_{\beta },\sigma_{\beta }^{2}\right)$. Further, with hyperpriors assumed for the hyperparameters μ_α_, μ_β_, $\sigma_{\alpha }^{2}$, $\sigma_{\beta }^{2}$, the joint posterior distribution of (θ, **ξ**, γ, **W, Z**, **μ**_ξ_, **Σ**_ξ_) is hence

(3) $$\begin{array}{l} p(\theta ,\xi ,\gamma ,W,Z,\mu_{\xi },\Sigma_{\xi }|y)\propto f(y|W,\gamma )p(W|Z)p(Z|\theta ,\xi )p(\theta )\\ \;p(\xi |\mu_{\xi },\Sigma_{\xi })p(\gamma )p(\mu_{\xi })p(\Sigma_{\xi }), \end{array}$$

where $\mu_{\xi }=\left(\mu_{\alpha },\mu_{\beta }\right)\prime ,\Sigma_{\xi }=\text{diag}\left(\sigma_{\alpha }^{2},\sigma_{\beta }^{2}\right),\text{ and }f\left(y|W,\gamma \right)={\prod \prod p_{ij}^{y_{ij}}\left(1-p_{ij}\right)}^{1-y_{ij}}$ is the likelihood function, with *p*_*ij*_ being the model probability function as defined in (1). Assume a normal prior for θ_*i*_, a conjugate Beta prior for γ_*j*_ so that $\theta_{i}\sim N\left(\mu ,\sigma^{2}\right)$, γ_*j*_ ~ *Beta*(*d, t*), and conditionally conjugate half-*t* prior distributions for σ_α_ and σ_β_ with mean 0, degrees-of-freedom ν and scale *s*, where ν and *s* are chosen to provide minimal prior information to constrain the scale hyperparameters to lie in a reasonable range. Hence, with the prior distributions specified this way, the full conditional distributions of all the parameters can be derived in closed forms through a multiplicative reparameterization following Knape et al. (2008) and updated iteratively using the Gibbs sampler (see the Appendix in Supplementary Material).

The half-*t* distribution considered for σ_α_ and σ_β_ takes the form

(4) $$\begin{array}{l} p\left(\sigma \right)\propto {\left(1+\frac{1}{\nu }{\left(\frac{\sigma }{s}\right)}^{2}\right)}^{-\frac{\nu +1}{2}} \end{array}$$

(Gelman, 2006). Setting ν = 1 results in a special case of half-Cauchy, which has a broad peak at zero and can be weakly informative if *s* takes large but finite values. On the other hand, setting the scale *s* to infinity corresponds to a flat prior distribution, setting the degrees of freedom ν to 100 corresponds to a half-normal distribution, which can be non-informative but proper if the scale *s* is set to a high value, such as 100. Here, in the study, we considered a half-normal, a half-Cauchy, and a half-*t* distribution with 4 degrees of freedom (ν = 4).

## 3. Methodology of Monte Carlo simulations

In order to evaluate the performance of the hierarchical 3PNO model as described in Section 2, two simulation studies were conducted where it was compared with other model specifications in parameter recovery and/or model comparisons.

### 3.1. Simulation study 1

In the IRT literature, it is well accepted that when prior information is not readily available, large data sizes are needed to estimate IRT parameters (e.g., Swaminathan and Gifford, 1983), as Bayesian estimation with a flat prior is equivalent to a maximum likelihood estimation, and that the accuracy of item (person) parameter estimates is related to the number of subjects (items) (e.g., Natesan et al., 2016; Sheng, 2010). Hence, in this simulation study, we evaluated the effect of sample size, test length, actual variability of item slope and intercept parameters on the accuracy with which model parameters are estimated considering a half-normal, a non-informative uniform or an inverse gamma prior.

Item responses for *k* items (*k* = 10, 20, and 40) and *n* individuals (*n* = 100, 300, 500, and 1000) were generated according to the 3PNO model, as defined in (1). Ability parameters were generated as samples from a standard normal distribution, pseudo-chance-level parameters were generated from a uniform distribution, γ_*j*_ ~ *U*(0.05, 0.4), and item slope and intercept parameters were generated as samples from uniform distributions so that

- Sim1: α_*j*_ ~ *U*(0, 2), β_*j*_ ~ *U*(−1, 1);
- Sim2: α_*j*_ ~ *U*(0, 2), β_*j*_ ~ *U*(−0.5, 0.5); and
- Sim3: α_*j*_ ~ *U*(0.5, 1.5), β_*j*_ ~ *U*(−1, 1).

When implementing the MCMC procedure, a diffuse prior were assumed for γ_*j*_, μ_α_ and μ_β_ so that γ_*j*_ ~ *Beta*(1, 1), *p*(μ_α_)∝1 and *p*(μ_β_)∝1. In addition, three ways of setting the prior distributions for α_*j*_ and β_*j*_ were considered such that both had

1. non-informative prior distributions that are uniform on σ, i.e., $p\left(\sigma_{\alpha }^{2}\right)\propto 1/\sigma_{\alpha }$ and $p\left(\sigma_{\beta }^{2}\right)\propto 1/\sigma_{\beta }$ ;
2. inverse-gamma (0.001, 0.001) prior distributions;
3. half-*t* prior distributions with 100 degrees of freedom, which are in practice equivalent to a half-normal.

It is noted that although the inverse-gamma (0.001, 0.001) prior density was not suggested by Gelman (2006), it was considered in this study because of its popularity (see e.g., Xu et al., 2009; O'Brien et al., 2015). With each of the prior specifications considered, the Gibbs sampling procedure was implemented where 10,000–50,000 iterations were obtained with the first half set as burn-in.

### 3.2. Simulation study 2

In the first simulation study, only two levels of variability (i.e., $\sigma_{\alpha ,\beta }^{2}=0.083$ and $\sigma_{\alpha ,\beta }^{2}=0.333$), and three different hyperpriors (one uniform, one inverse-gamma and one half-normal) were considered. Given that the range for item intercept parameters (β_*j*_) is generally wider than [−1, 1] in practice, it would be interesting to see how the weakly informative half-*t* family performs when the variance for β goes beyond 1. Hence, a second simulation study was conducted where two factors were manipulated, namely, actual variability of the item intercept parameters and specifications of prior distributions for the item scale hyperparameters.

Item responses for 20 items and 1000 individuals were generated according to the 3PNO model, as defined in (1). Ability parameters were generated as samples from a standard normal distribution, item slope and pseudo-chance-level parameters were generated from uniform distributions, α_*j*_ ~ *U*(0, 2) and γ_*j*_ ~ *U*(0.05, 0.4), and item intercept parameters were generated as samples from uniform distributions so that

- Sim1: β ~ *U*(−0.5, 0.5);
- Sim2: β ~ *U*(−1, 1);
- Sim3: β ~ *U*(−2, 2); and
- Sim4: β ~ *U*(−4, 4).

It is noted that in the four simulations, the uniform distributions from which the intercept parameters were sampled from have an increasingly large σ_β_ ranging from 0.29−2.31 (with the corresponding variance ranging from 0.083−5.333).

In addition, six ways of setting the prior distributions for $\sigma_{\alpha }^{2}$ and $\sigma_{\beta }^{2}$ were considered such that both had

1. non-informative prior distributions that are uniform on σ, i.e., $p\left(\sigma_{\alpha }^{2}\right)\propto 1/\sigma_{\alpha }$ and $p\left(\sigma_{\beta }^{2}\right)\propto 1/\sigma_{\beta }$;
2. non-informative inverse-gamma (0.001, 0.001) prior distributions for σ^2^;
3. informative inverse-gamma (3, 2) prior distributions for σ^2^;
4. weakly informative half-Cauchy prior distributions for σ;
5. weakly informative half-*t* prior distributions for σ with ν = 100, which in practice are equivalent to half-normal distributions;
6. weakly informative half-*t* prior distributions for σ with ν = 4.

The Gibbs sampling procedure was implemented where 20,000 iterations were obtained with the first 10,000 as burn-in.

### 3.3. Evaluation criteria

**Figure 1:**
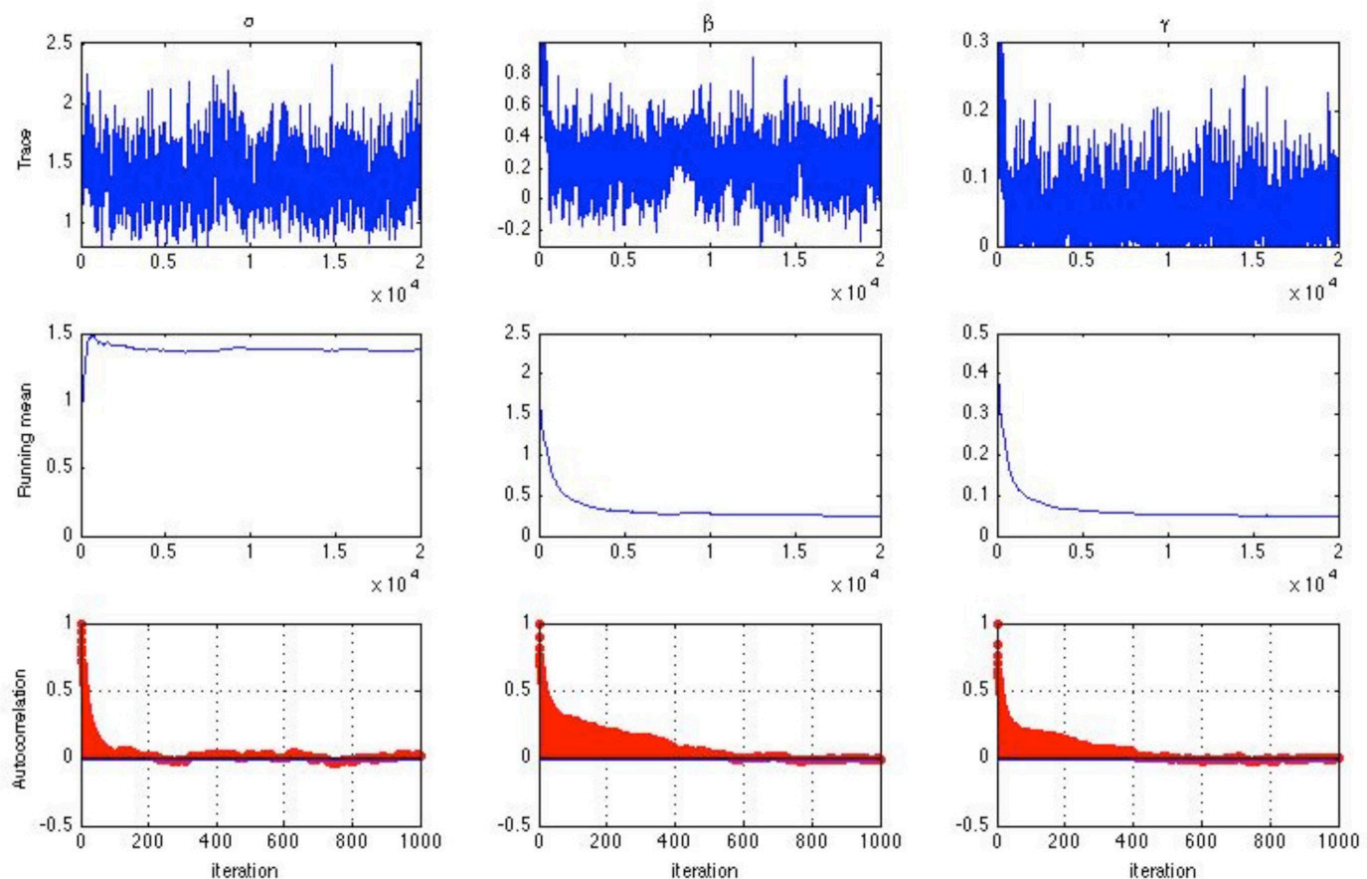
**Trace plots (top)**, running mean plots **(middle)** and autocorrelation plots **(bottom)** of α, β and γ for one item assuming a half-normal prior distribution for σ_α_ and σ_β_ [*n* = 1000, *k* = 10, chain-length = 20, 000, item slopes and intercepts were generated from α_*j*_ ~ *U*(0, 2) and β_*j*_ ~ *U*(−1, 1)].

For both simulation studies, convergence was evaluated using the Gelman-Rubin R (Gelman and Rubin, 1992) statistic. The usual practice is using multiple Markov chains from different starting points. Alternatively, a single chain can be divided into sub-chains so that convergence is assessed by comparing the between and within sub-chain variance (Fox, 2007). Since a single chain is less wasteful in the number of iterations needed, the latter approach was adopted. For each Markov chain, the initial values were set to be α_*j*_ = 1, β_*j*_ = 0, and γ_*j*_ = 0.2 for all items *j* and θ_*i*_ = 0 for all persons *i*. After discarding the burn-in samples, the chain was then separated into five sub-chains of equal length and the R statistic was calculated following the procedure by Gelman and Rubin (1992). Convergence can also be monitored visually using time series graphs of the simulated sequence, such as the trace plot, the running mean plot, and the autocorrelation plot shown in Figure 1 for one item. It is observed that for this item, the autocorrelations between successive parameter draws became negligible at lags > 600, suggesting burn-in for a single chain should not take longer than that (Geyer, 1992). Indeed, the trace plot and the running mean plot both suggest possible convergence within 10,000 iterations. Inspection of such plots has, however, been criticized for being unreliable and unwieldy in the presence of a large number of model parameters (Gelman et al., 2003; Nylander et al., 2008). The R statistic obtained from using a single chain was hence the major approach for assessing convergence in this study.

For each simulated scenario, 25 replications, as recommended by Harwell et al. (1996), were conducted to avoid erroneous results in estimation due to sampling error. The accuracy of item/person parameter estimates was evaluated using the root mean square error (*RMSE*) and bias. Let τ denote the true value of a parameter (e.g., α_*j*_, β_*j*_, γ_*j*_, or θ_*i*_) and *t*_*r*_ its estimate in the *r*th replication (*r* = 1, …, *R*). The *RMSE* is defined as

(5) $$\begin{array}{l} RMSE_{\tau }=\sqrt{\frac{\sum_{r\;=\;1}^{R}{\left(t_{r}-\tau \right)}^{2}}{R}}, \end{array}$$

and the *bias* is defined as

(6) $$\begin{array}{l} bias_{\tau }=\frac{\sum_{r\;=\;1}^{R}\left(t_{r}-\tau \right)}{R}. \end{array}$$

These quantities were averaged over items/persons to provide summary indices.

In simulation study 2 where six model specifications were compared, the adequacy of the fit of the hierarchical 3PNO model with a given prior density on the simulated data was evaluated using Bayesian deviance. It should be noted that this measure provides a model comparison criterion. Hence, it evaluates the fit of a model in a relative, not absolute, sense. The Bayesian deviance information criterion (DIC) was introduced by Spiegelhalter et al. (2002) who generalized the classical information criteria (e.g., AIC, BIC) to one that is based on the posterior distribution of the deviance. This criterion is defined as $DIC=\bar{D}+p_{D}$, where $\bar{D}=E\left[-2\log L_{\vartheta |y}\left(y|\vartheta \right)\right]$ is the posterior expectation of the deviance (with *L* being the likelihood function), and $p_{D}=E_{\vartheta |y}\left(D\right)-D\left[E_{\vartheta |y}\left(\vartheta \right)\right]=\bar{D}-D\left(\bar{\vartheta }\right)$ is the effective number of parameters (Carlin and Louis, 2000). In addition, let $D\left(\bar{\vartheta }\right)=-2\log\left[L_{\vartheta |y}\left(\text{y}|\bar{\vartheta }\right)\right]$, where $\bar{\vartheta }$ is the posterior mean. To compute Bayesian DIC, MCMC samples of the parameters, ϑ^(1)^, …, ϑ^(*G*)^, can be drawn from the Gibbs sampler, then $\bar{D}$ is approximated as $\bar{D}=\frac{1}{G}\left[-2\log\prod_{g\;=\;1}^{G}L\left(\text{y}|\vartheta^{\left(g\right)}\right)\right]$. Generally more complicated models tend to provide better fit. Hence, penalizing for the number of parameters makes DIC a more reasonable measure to use. In this study, the Bayesian deviance estimate was obtained after each implementation and averaged across the 25 replications for each model specification.

## 4. Simulation results

The results of the two simulation studies are presented in this section and described as follows.

### 4.1. Simulation study 1

Each implementation of the Gibbs sampler gave rise to Gelman-Rubin R statistics close to 1, indicating possible convergence of the Markov chains within the simulated number of iterations. Hence, the posterior estimates were obtained as the posterior expectations of the Gibbs samples and the average *RMSE* and *bias* values for α_*j*_, β_*j*_, γ_*j*_, and θ_*i*_ are summarized in Tables 1–4. A close examination of these values leads to the following observations:

1. In Sim1 where the actual variances for α and β were both 0.333, the half-normal distribution performed relatively less well in recovering item and person parameters than the uniform or inverse-gamma distributions when data sizes (*n* × *k*) were < 10,000, although it has to be noted that this distribution consistently resulted in smaller *bias* in estimating α regardless of sample size or test length. The uniform prior density performed similarly as the inverse-gamma prior density, with a slight advantage for data with small sample sizes (i.e., *n* = 100) especially where *k* = 10.
2. Sim2 differed from Sim1 in that the actual variance for β decreased to 0.083, a value closer to zero. Hence, we focus on the results in recovering β here. From Table 2, it is obvious that the half-normal distribution consistently performed better in recovering β than the uniform or inverse-gamma distribution when *k* < 40 or with large sample sizes (*n* > 300) when *k* = 40. Between the two non-informative priors, the uniform prior density performed less well than the inverse-gamma (0.001, 0.001) prior density.
3. Similar to Sim2, Sim3 differed from Sim1 in the actual variance for α being reduced to 0.083, and consequently, the results in recovering α are discussed here. It is noted from Table 1 that the half-normal distribution consistently resulted in smaller *RMSE* and *bias* and hence performed better than the non-informative uniform or inverse-gamma family of prior distributions (except for the situations where *n* < 500 and *k* = 40). The uniform prior density tended to result in larger *RMSE* or *bias* than the inverse-gamma (0.001, 0.001) prior when *k* < 40.
4. It is interesting to note that the half-normal prior density consistently resulted in smaller bias in estimating α in all the simulated conditions.
5. Further, it is noted that the benefit of using a half-normal prior in Sim2 or Sim3 where it resulted in smaller *RMSE* and *bias* in estimating β or α was not reflected in estimating θ unless the data size was over 12, 000 in Sim2 or unless *k* ≥ 20 in Sim3.

**Table 1:** **Average ***RMSE*** and ***bias*** for recovering slope (α) parameters in the hierarchical 3PNO model with the three prior specifications under the three test length, four sample size and three actual variance conditions**.

		***RMSE***			***bias***		
	***n***	**uniform**	**inv-g**	**half-n**	**uniform**	**inv-g**	**half-n**
		**Sim1:** α ~ *U*(0, 2), β ~ *U*(−1, 1)					
*k* = 10	100	0.3213	0.3737	0.2686	0.3544	0.3810	0.0959
	300	0.1103	0.1082	0.1422	0.0976	0.0889	0.0704
	500	0.0865	0.0868	0.1271	0.1046	0.0957	0.0656
	1000	0.0780	0.0764	0.0964	0.0814	0.0716	0.0481
*k* = 20	100	0.1656	0.1665	0.1766	0.0617	0.0604	0.0589
	300	0.1142	0.1157	0.1029	0.0737	0.0768	0.0613
	500	0.0831	0.0843	0.0634	0.0627	0.0673	0.0285
	1000	0.0705	0.0721	0.0521	0.0535	0.0568	0.0432
*k* = 40	100	0.2238	0.2253	0.1401	0.1834	0.1852	0.0584
	300	0.1863	0.1877	0.0840	0.2017	0.2034	0.0441
	500	0.1604	0.1624	0.0550	0.2034	0.2071	0.0390
	1000	0.1339	0.1358	0.0366	0.1890	0.1915	0.0372
		**Sim2:** α ~ *U*(0, 2), β ~ *U*(−0.5, 0.5)					
*k* = 10	100	0.2690	0.2350	0.2491	0.3179	0.2743	0.0668
	300	0.0962	0.0929	0.0724	0.1625	0.1492	0.0361
	500	0.0687	0.0709	0.0517	0.1257	0.1227	0.0255
	1000	0.0534	0.0542	0.0539	0.0577	0.0518	0.0282
*k* = 20	100	0.1558	0.1565	0.1503	0.0784	0.0746	0.0546
	300	0.0901	0.0914	0.0641	0.0401	0.0420	0.0357
	500	0.0775	0.0787	0.0500	0.0614	0.0656	0.0335
	1000	0.0502	0.0508	0.0319	0.0528	0.0550	0.0282
*k* = 40	100	0.2094	0.2112	0.1313	0.1623	0.1655	0.1020
	300	0.1500	0.1511	0.0547	0.1918	0.1938	0.0373
	500	0.1372	0.1381	0.0449	0.1876	0.1877	0.0413
	1000	0.1141	0.1151	0.0254	0.1842	0.1857	0.0300
		**Sim3:** α ~ *U*(0.5, 1.5), β ~ *U*(−1, 1)					
*k* = 10	100	0.3572	0.3359	0.1882	0.5038	0.4847	0.2929
	300	0.1237	0.1230	0.0860	0.2159	0.2145	0.0890
	500	0.0785	0.0745	0.0725	0.1658	0.1603	0.0749
	1000	0.0634	0.0650	0.0537	0.1128	0.1092	0.0452
*k* = 20	100	0.1042	0.1063	0.0852	0.1780	0.1789	0.0905
	300	0.0715	0.0709	0.0675	0.1080	0.1061	0.0771
	500	0.0516	0.0511	0.0506	0.0820	0.0797	0.0679
	1000	0.0430	0.0426	0.0348	0.0440	0.0439	0.0421
*k* = 40	100	0.0687	0.0688	0.0763	0.0506	0.0501	0.0590
	300	0.0499	0.0500	0.0531	0.0371	0.0366	0.0599
	500	0.0506	0.0507	0.0404	0.0418	0.0436	0.0391
	1000	0.0420	0.0419	0.0250	0.0400	0.0390	0.0261

*inv-g, inverse-gamma; half-n, half-normal*.

**Table 2:** **Average ***RMSE*** and ***bias*** for recovering intercept (β) parameters in the hierarchical 3PNO model with the three prior specifications under the three test length, four sample size and three actual variance conditions**.

		***RMSE***			***bias***		
	***n***	**uniform**	**inv-g**	**half-n**	**uniform**	**inv-g**	**half-n**
		**Sim1:** α ~ *U*(0, 2), β ~ *U*(−1, 1)					
*k* = 10	100	0.4967	0.6177	0.4233	0.5017	0.5509	0.3778
	300	0.2153	0.2088	0.2642	0.2329	0.2208	0.2231
	500	0.1960	0.1939	0.2532	0.2096	0.2011	0.2015
	1000	0.1599	0.1580	0.1757	0.1550	0.1436	0.1179
*k* = 20	100	0.3474	0.3458	0.4409	0.3528	0.3504	0.3864
	300	0.1906	0.1874	0.2047	0.1832	0.1781	0.2074
	500	0.1325	0.1334	0.1307	0.1188	0.1188	0.1466
	1000	0.1590	0.1590	0.1545	0.1331	0.1312	0.1602
*k* = 40	100	0.3184	0.3189	0.3748	0.2940	0.2942	0.3645
	300	0.2283	0.2288	0.2042	0.1818	0.1818	0.2185
	500	0.1852	0.1846	0.1228	0.1281	0.1240	0.1383
	1000	0.1550	0.1555	0.1162	0.0955	0.0946	0.1243
		**Sim2:** α ~ *U*(0, 2), β ~ *U*(−0.5, 0.5)					
*k* = 10	100	0.3842	0.3199	0.1581	0.4892	0.4443	0.2364
	300	0.2073	0.1995	0.0884	0.2937	0.2816	0.1186
	500	0.1573	0.1580	0.0892	0.2281	0.2282	0.1225
	1000	0.1108	0.1050	0.0600	0.1631	0.1563	0.0711
*k* = 20	100	0.1621	0.1589	0.1436	0.2864	0.2823	0.2325
	300	0.0956	0.0937	0.0894	0.1724	0.1673	0.1475
	500	0.0889	0.0861	0.0714	0.1341	0.1316	0.1115
	1000	0.0536	0.0554	0.0400	0.0782	0.0792	0.0573
*k* = 40	100	0.1192	0.1188	0.1448	0.2338	0.2304	0.2508
	300	0.0634	0.0633	0.0788	0.0831	0.0834	0.1295
	500	0.0617	0.0620	0.0616	0.0650	0.0664	0.0920
	1000	0.0507	0.0506	0.0388	0.0485	0.0490	0.0593
		**Sim3:** α ~ *U*(0.5, 1.5), β ~ *U*(−1, 1)					
*k* = 10	100	0.4323	0.4214	0.6113	0.4942	0.4832	0.5111
	300	0.1668	0.1702	0.2306	0.2299	0.2343	0.2255
	500	0.0966	0.0961	0.1539	0.1595	0.1560	0.1658
	1000	0.0756	0.0790	0.0879	0.0882	0.0859	0.0794
*k* = 20	100	0.3288	0.3351	0.4090	0.3854	0.3864	0.3921
	300	0.1613	0.1635	0.1348	0.1932	0.1940	0.1898
	500	0.1192	0.1206	0.0999	0.1394	0.1395	0.1461
	1000	0.0765	0.0773	0.0567	0.0684	0.0699	0.0761
*k* = 40	100	0.3336	0.3331	0.2748	0.3281	0.3260	0.2882
	300	0.1999	0.2039	0.1074	0.1863	0.1930	0.1494
	500	0.1585	0.1591	0.0748	0.1346	0.1335	0.0966
	1000	0.1158	0.1162	0.0429	0.0827	0.0835	0.0505

*inv-g, inverse-gamma; half-n, half-normal*.

**Table 3:** **Average ***RMSE*** and ***bias*** for recovering guessing (γ) parameters in the hierarchical 3PNO model with the three prior specifications under the three test length, four sample size and three actual variance conditions**.

		***RMSE***			***bias***		
	***n***	**uniform**	**inv-g**	**half-n**	**uniform**	**inv-g**	**half-n**
		**Sim1:** α ~ *U*(0, 2), β ~ *U*(−1, 1)					
*k* = 10	100	0.0357	0.0371	0.0407	0.0990	0.1026	0.0964
	300	0.0306	0.0304	0.0359	0.0848	0.0826	0.0831
	500	0.0294	0.0290	0.0338	0.0752	0.0733	0.0701
	1000	0.0278	0.0271	0.0289	0.0712	0.0677	0.0608
*k* = 20	100	0.0443	0.0444	0.0509	0.1111	0.1110	0.1192
	300	0.0301	0.0300	0.0306	0.0664	0.0652	0.0730
	500	0.0244	0.0245	0.0239	0.0539	0.0536	0.0631
	1000	0.0305	0.0305	0.0284	0.0659	0.0651	0.0728
*k* = 40	100	0.0346	0.0346	0.0356	0.0796	0.0794	0.0881
	300	0.0329	0.0330	0.0300	0.0557	0.0557	0.0688
	500	0.0271	0.0270	0.0207	0.0386	0.0379	0.0497
	1000	0.0273	0.0273	0.0210	0.0372	0.0368	0.0493
		**Sim2:** α ~ *U*(0, 2), β ~ *U*(−0.5, 0.5)					
*k* = 10	100	0.0298	0.0286	0.0227	0.1128	0.1082	0.0740
	300	0.0220	0.0217	0.0148	0.0855	0.0831	0.0425
	500	0.0190	0.0190	0.0159	0.0718	0.0716	0.0452
	1000	0.0158	0.0157	0.0125	0.0553	0.0534	0.0301
*k* = 20	100	0.0195	0.0193	0.0186	0.0786	0.0776	0.0641
	300	0.0158	0.0158	0.0153	0.0599	0.0588	0.0508
	500	0.0155	0.0151	0.0130	0.0458	0.0450	0.0373
	1000	0.0098	0.0102	0.0080	0.0335	0.0341	0.0264
*k* = 40	100	0.0161	0.0160	0.0172	0.0477	0.0469	0.0498
	300	0.0117	0.0117	0.0119	0.0206	0.0208	0.0324
	500	0.0117	0.0118	0.0108	0.0204	0.0206	0.0296
	1000	0.0103	0.0103	0.0076	0.0153	0.0153	0.0190
		**Sim3:** α ~ *U*(0.5, 1.5), β ~ *U*(−1, 1)					
*k* = 10	100	0.0397	0.0394	0.0557	0.1301	0.1287	0.1505
	300	0.0254	0.0258	0.0318	0.0825	0.0838	0.0812
	500	0.0164	0.0162	0.0227	0.0605	0.0596	0.0604
	1000	0.0160	0.0169	0.0182	0.0480	0.0482	0.0445
*k* = 20	100	0.0374	0.0377	0.0441	0.1129	0.1132	0.1207
	300	0.0235	0.0236	0.0196	0.0698	0.0700	0.0664
	500	0.0220	0.0221	0.0181	0.0641	0.0639	0.0613
	1000	0.0157	0.0157	0.0118	0.0418	0.0420	0.0382
*k* = 40	100	0.0359	0.0359	0.0328	0.0908	0.0905	0.0874
	300	0.0246	0.0248	0.0164	0.0541	0.0551	0.0505
	500	0.0223	0.0224	0.0129	0.0465	0.0463	0.0399
	1000	0.0178	0.0179	0.0089	0.0330	0.0336	0.0253

*inv-g, inverse-gamma; half-n, half-normal*.

**Table 4:** **Average ***RMSE*** and ***bias*** for recovering person parameters (θ) in the hierarchical 3PNO model with the three prior specifications under the three test length, four sample size and three actual variance conditions**.

		***RMSE***			***bias***		
	***n***	**uniform**	**inv-g**	**half-n**	**uniform**	**inv-g**	**half-n**
		**Sim1:** α ~ *U*(0, 2), β ~ *U*(−1, 1)					
*k* = 10	0100	0.3618	0.3626	0.3951	0.1188	0.1208	0.1257
	300	0.3240	0.3239	0.3331	0.1011	0.1009	0.1041
	500	0.3045	0.3047	0.3098	0.0952	0.0950	0.0959
	1000	0.3003	0.3005	0.3024	0.0908	0.0909	0.0902
*k* = 20	100	0.2239	0.2239	0.2403	0.0989	0.0981	0.1208
	300	0.2072	0.2071	0.2079	0.0882	0.0882	0.0857
	500	0.1919	0.1922	0.1895	0.0733	0.0737	0.0706
	1000	0.1945	0.1947	0.1927	0.0703	0.0706	0.0686
*k* = 40	100	0.1827	0.1827	0.1774	0.1450	0.1468	0.1553
	300	0.1648	0.1650	0.1412	0.1196	0.1201	0.0766
	500	0.1483	0.1485	0.1236	0.1140	0.1132	0.0602
	1000	0.1430	0.1434	0.1263	0.0858	0.0865	0.0562
		**Sim2:** α ~ *U*(0, 2), β ~ *U*(−0.5, 0.5)					
*k* = 10	100	0.3309	0.3309	0.3480	0.0971	0.0976	0.1031
	300	0.3225	0.3220	0.3274	0.0941	0.0937	0.0981
	500	0.3050	0.3051	0.3073	0.0844	0.0846	0.0851
	1000	0.3001	0.3000	0.3015	0.0857	0.0858	0.0866
*k* = 20	100	0.2122	0.2122	0.2200	0.0712	0.0714	0.0871
	300	0.2036	0.2038	0.2063	0.0786	0.0787	0.0864
	500	0.1932	0.1932	0.1950	0.0728	0.0728	0.0771
	1000	0.1885	0.1885	0.1879	0.0653	0.0655	0.0652
*k* = 40	100	0.1685	0.1691	0.1696	0.1303	0.1300	0.1553
	300	0.1429	0.1435	0.1341	0.0804	0.0812	0.0796
	500	0.1384	0.1384	0.1287	0.0709	0.0710	0.0613
	1000	0.1365	0.1370	0.1269	0.0643	0.0647	0.0568
		**Sim3:** α ~ *U*(0.5, 1.5), β ~ *U*(−1, 1)					
*k* = 10	100	0.3341	0.3337	0.3562	0.1101	0.1115	0.1139
	300	0.3035	0.3034	0.3090	0.0883	0.0879	0.0909
	500	0.2924	0.2923	0.2979	0.0915	0.0912	0.0916
	1000	0.2933	0.2934	0.2944	0.0857	0.0859	0.0864
*k* = 20	100	0.1983	0.1989	0.2068	0.0771	0.0772	0.0759
	300	0.1920	0.1918	0.1886	0.0760	0.0762	0.0691
	500	0.1877	0.1878	0.1867	0.0679	0.0682	0.0664
	1000	0.1796	0.1798	0.1779	0.0677	0.0680	0.0667
*k* = 40	100	0.1434	0.1427	0.1302	0.0989	0.0979	0.0729
	300	0.1367	0.1374	0.1224	0.0992	0.1014	0.0553
	500	0.1272	0.1276	0.1155	0.0840	0.0840	0.0540
	1000	0.1200	0.1199	0.1120	0.0695	0.0692	0.0532

*inv-g, inverse-gamma; half-n, half-normal*.

From these observations, it is hence noted that the half-normal prior outperforms the uniform or inverse-gamma family of prior distributions in estimating the corresponding parameters in the hierarchical 3PNO model when the variability for item slope or intercept parameters is close to zero. Further, the inverse-gamma (0.001, 0.001) prior tends to perform better in parameter recovery than the uniform prior when the respective item parameters have a small variance. This can be explained by the fact that the non-informative uniform prior density is flat and hence does not restrict σ away from large values, whereas inverse-gamma (0.001, 0.001) and half-normal distributions have a peak around zero and do perform such shrinkage for variances near zero. It is further noted that the estimation error and bias in estimating item parameters (α, β, or γ) reduce with the increase of sample sizes, and that the error and bias in estimating person parameters (θ) reduce with the increase of test lengths. This is consistent with findings from other studies (e.g., Swaminathan and Gifford, 1983; Sheng, 2010; Natesan et al., 2016).

### 4.2. Simulation study 2

Each implementation of the Gibbs sampler gave rise to Gelman-Rubin R statistics close to 1, indicating possible convergence within 20,000 iterations. Hence, the posterior estimates were obtained as the posterior expectations of the Gibbs samples and the average *RMSE* and *bias* values for α_*j*_, β_*j*_, and γ_*j*_ are summarized in Table 5. A close examination of these values leads to the following observations:

1. In all four simulations where the actual variability for the intercept parameters (σ_β_) ranged between 0.29−2.31, the three weakly informative half-*t* prior densities, namely, the half-Cauchy, the half-normal and the half-*t* with 4 degrees of freedom had consistently smaller *RMSE* if not *bias* in recovering item and person parameters, and in particular in recovering β_*j*_, compared with the other three prior distributions.
2. It is noted that in Sim2 where σ_β_ was about 0.5, the three weakly informative half-*t* densities had more bias in estimating β and γ than the two non-informative prior densities. When σ_β_ moved further away from 0.5 (i.e., toward 0 in Sim1 or toward 2.5 in Sim4), their advantages over other model specifications became more obvious in that they resulted in much smaller average *RMSE* and *bias* values.
3. Among the three weakly informative half-*t* densities, the half-normal resulted in relatively smaller *RMSE* and *bias* in recovering item and person parameters in Sim1, but larger *RMSE* and *bias* in Sim2, Sim3, and Sim4.
4. Between the two non-informative priors, the inverse-gamma (0.001, 0.001) prior distribution performed slightly better in estimating β and γ in Sim1, but worse in estimating other parameters or in other situations. This is consistent with findings from the first simulation study.
5. In Sim1 where σ_β_ was close to 0, the informative inverse-gamma (3, 2) prior distribution was correctly specified with little bias and hence had small *RMSE* in recovering β_*j*_. However, when σ_β_ moved further away from 0, it became less appropriate, and consequently resulted in an obviously larger bias and *RMSE* in recovering especially item parameters.

**Table 5:** **Average ***RMSE*** and ***bias*** for recovering item and person parameters in the hierarchical 3PNO model with the six prior specifications (*n* = 1000, *k* = 20)**.

		***RMSE***				***bias***			
	***prior*_*αβ*_**	**Sim1**	**Sim2**	**Sim3**	**Sim4**	**Sim1**	**Sim2**	**Sim3**	**Sim4**
α	1	0.0550	0.0709	0.1609	0.3455	0.0493	0.0774	0.0868	0.2735
	2	0.0566	0.0730	0.1612	0.3502	0.0515	0.0798	0.0904	0.2810
	3	0.1011	0.1198	0.1950	0.3825	0.1450	0.1432	0.1566	0.3528
	4	0.0388	0.0487	0.0967	0.1588	0.0407	0.0550	0.0843	0.0654
	5	0.0365	0.0524	0.1016	0.2054	0.0265	0.0419	0.0392	0.1241
	6	0.0407	0.0489	0.0936	0.1620	0.0405	0.0500	0.0796	0.0550
β	1	0.0625	0.1302	0.6378	4.0725	0.0744	0.0987	0.1819	0.4088
	2	0.0607	0.1316	0.6450	4.0914	0.0727	0.0997	0.1836	0.4067
	3	0.0514	0.1655	0.8096	4.5642	0.0327	0.1007	0.2275	0.4900
	4	0.0453	0.1206	0.3886	1.2150	0.0569	0.1334	0.2348	0.2623
	5	0.0451	0.1230	0.3687	2.0226	0.0467	0.1126	0.1905	0.2543
	6	0.0482	0.1200	0.3653	1.2272	0.0569	0.1242	0.2276	0.2413
γ	1	0.0099	0.0235	0.0793	0.1666	0.0278	0.0445	0.0988	0.1811
	2	0.0096	0.0238	0.0798	0.1672	0.0267	0.0449	0.0994	0.1806
	3	0.0093	0.0275	0.0889	0.1779	0.0139	0.0426	0.1069	0.1940
	4	0.0075	0.0198	0.0493	0.0774	0.0241	0.0503	0.0915	0.1289
	5	0.0078	0.0218	0.0560	0.1002	0.0211	0.0479	0.0926	0.1361
	6	0.0079	0.0199	0.0489	0.0784	0.0242	0.0481	0.0910	0.1286
θ	1	0.2007	0.2010	0.2266	0.3273	0.0722	0.0731	0.0883	0.0973
	2	0.2009	0.2011	0.2274	0.3280	0.0722	0.0733	0.0890	0.0974
	3	0.2032	0.2078	0.2408	0.3521	0.0739	0.0802	0.1017	0.1034
	4	0.2003	0.1978	0.2097	0.2812	0.0716	0.0706	0.0707	0.0809
	5	0.2004	0.1987	0.2106	0.2865	0.0718	0.0711	0.0713	0.0814
	6	0.2004	0.1976	0.2095	0.2822	0.0718	0.0706	0.0709	0.0812

*Sim1, β ~ U(−0.5, 0.5); Sim2, β ~ U(−1, 1); Sim3, β ~ U(−2, 2); Sim4, β ~ U(−4, 4)*.

**Figure 2:**
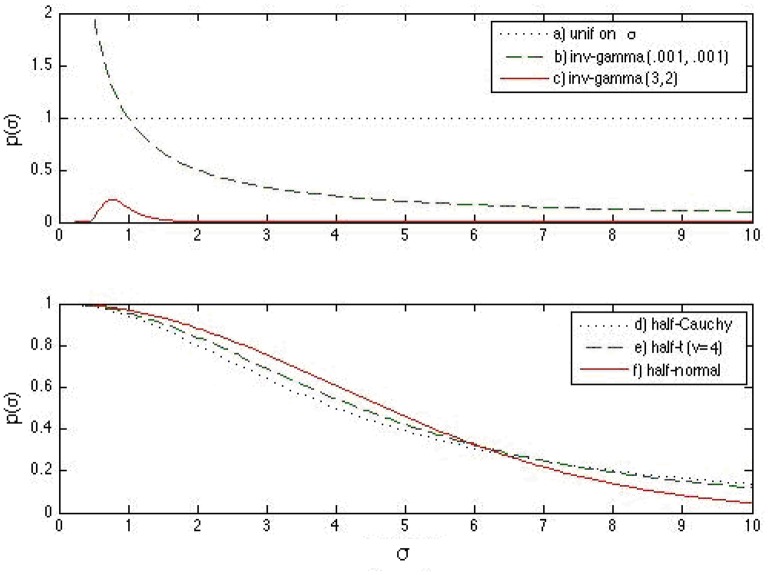
**Prior density functions for the slope and intercept standard deviation parameters: (a)** uniform prior distribution on σ_α_ (or σ_β_), **(b)** inverse-gamma (0.001, 0.001) prior distribution on $\sigma_{\alpha }^{2}$ (or $\sigma_{\beta }^{2}$), **(c)** inverse-gamma (3, 2) prior distribution on $\sigma_{\alpha }^{2}$ (or $\sigma_{\alpha }^{2}$), **(d)** half-Cauchy prior distribution for σ_α_ (or σ_β_), **(e)** half-*t* prior distribution for σ_α_ (or σ_β_) with 4 degrees of freedom, and **(f)** half-normal prior distribution for σ_α_ (or σ_β_). It is noted that the half-*t* distributions are on a different scale compared to the previous three densities.

From these observations, it is noted that the weakly informative half-*t* family works well in a wider range of situations than the non-informative or the informative prior densities in recovering the 3PNO model item parameters. Specifically, when the actual scale hyperparameter is close to 0, the non-informative prior density does not work well compared with informative or weakly informative prior densities, as prior information helps to restrict σ_β_ away from large values. Even with prior information, the informative inverse-gamma (3,2) prior density does not outperform the weakly informative half-*t* distribution because the latter has a better behavior near 0. Figure 2 graphically illustrates this point. The non-informative inverse-gamma prior distribution, in general, is not recommended for large σ values because it is not non-informative for these values (see Figure 2). On the other hand, prior information has to be correctly specified, as misspecification leads to large bias and hence large estimation error, e.g., the actual σ_β_ in Sim4 was clearly not in the range of the inverse-gamma (3, 2) distribution (see Figure 2). When comparing among the three half-*t* distributions, the half-Cauchy is more likely to allow for occasionally large values than the half-*t* density with 4 degrees of freedom, or the half-normal, which has the smallest tail (see Figure 2). Hence, if it is known a priori that the scale hyperparameter might be far away from 0, a half-Cauchy or a half-*t* distribution with a small degrees of freedom is suggested. Otherwise, a half-normal may be considered.

In addition to parameter recovery, the model comparison results in each simulation were averaged over the 25 replications and are summarized in Table 6, which shows the averaged estimates for the posterior expectation of the deviance ($\bar{D}$), the deviance of the posterior expectation [$D\left(\bar{\vartheta }\right)$] values, the effective number of parameters (*p*_*D*_), and the Bayesian *DIC*, respectively. The model with the half-Cauchy or half-*t* prior density shows consistently smaller $\bar{D}$, $D\left(\bar{\vartheta }\right)$, and *DIC* than those with other prior specifications. Since small deviance values indicate better model fit, models with such prior distributions for the scale hyperparameters are shown to provide a better description of the simulated data when the actual σ_β_ was especially larger than 0.29, even after penalizing for model complexities, i.e., the effective number of parameters.

**Table 6:** **Average Bayesian deviance estimates for the hierarchical 3PNO model with the six prior specifications under four simulated scenarios (***n*** = 1000, ***k*** = 20)**.

	***prior*_*αβ*_**	**$\bar{D}$**	**$D\left(\bar{\vartheta }\right)$**	***p*_*D*_**	***DIC***
Sim1	1	21441.66	20595.02	846.65	22288.31
	2	21444.21	20597.86	846.34	22290.55
	3	21499.50	20651.26	848.23	22347.73
	4	21420.74	20589.93	830.81	22251.54
	5	21434.56	20598.11	836.45	22271.01
	6	21421.36	20589.28	832.08	22253.44
Sim2	1	20793.37	19947.51	845.86	21639.23
	2	20795.94	19950.44	845.50	21641.43
	3	20881.40	20045.02	836.39	21717.79
	4	20730.70	19888.92	841.78	21572.48
	5	20752.65	19906.82	845.83	21598.49
	6	20731.41	19888.26	843.15	21574.56
Sim3	1	18543.51	17741.73	801.79	19345.30
	2	18547.95	17746.12	801.82	19349.77
	3	18686.54	17908.49	778.04	19464.58
	4	18310.29	17473.68	836.60	19146.89
	5	18347.90	17514.27	833.63	19181.54
	6	18313.22	17477.57	835.65	19148.87
Sim4	1	15932.74	15262.86	669.88	16602.62
	2	15940.13	15270.32	669.82	16609.95
	3	16109.74	15489.93	619.81	16729.55
	4	15510.46	14766.48	743.98	16254.44
	5	15581.47	14850.04	731.43	16312.89
	6	15512.23	14773.10	739.13	16251.36

Sim1, β ~ U(−0.5, 0.5); Sim2, β ~ U(−1, 1); Sim3, β ~ U(−2, 2); Sim4, β ~ U(−4, 4)

After a close examination and comparison of the values shown in the table, a few remarks can be drawn from these results:

- In all four simulations where σ_β_ ranged between 0.29−2.31, Bayesian deviances consistently preferred the models with half-*t* prior densities, whose deviance values were increasingly smaller compared with other model specifications when σ_β_ became larger.
- The two non-informative priors performed similarly in describing the simulated data according to Bayesian DIC, which was slightly larger for the model with the inverse-gamma (0.001, 0.001) prior.
- In all the simulated scenarios, the model with the informative inverse-gamma (3,2) prior distribution had consistently the largest average deviance values and hence is not preferred. One may further note that as σ_β_ moved further away from 0, the difference in deviances between this model specification and others was increasingly larger.
- Among the three half-*t* distributions considered, the Bayesian deviance measures did not favor the half-normal prior density.
- It is interesting to note that when σ_β_ was small with values of e.g., 0−0.6, models with half-*t* prior densities tended to have smaller effective number of parameters (*p*_*D*_) than those with non-informative prior densities. The opposite is true for situations where σ_β_ was over 1.

In summary, the results in parameter recovery and model comparisons using Bayesian deviances suggest that the hierarchical 3PNO model with weakly informative half-*t* densities for the item scale hyperparameters does show advantages compared with those with non-informative or informative prior distributions in various test situations where the actual variability ranges from small to large values. When the actual variability of item parameters is not close to 0, the half-Cauchy or the half-*t* with small degrees of freedom is preferred over the half-normal distribution because of its flexibility in allowing for occasional large variability.

## 5. An example with *CBASE* data

As an illustration, the hierarchical 3PNO model with the weakly informative half-*t* hyperpior was implemented to a subset of *CBASE English* subject data, and further compared with the other model specifications in describing the data.

### 5.1. Method

The overall *CBASE* exam contains 25 multiple-choice items on English reading/literature. The data used in this study were from college students who took the LP form of *CBASE* in years 2001 and 2002. After removing those who attempted the exam multiple times and removing missing responses, a sample of 1,200 examinees was randomly selected. To assess the model goodness-of-fit, hierarchical 3PNO models with the three half-*t* prior distributions were compared with each other and further compared with those with uniform and inverse-gamma prior densities described in Section 4.

### 5.2. Results

Each of the six model specifications was implemented to the *CBASE* English data using the Gibbs sampling procedure, where 20,000 iterations were obtained with the first 10,000 set as burn-in. The Gelman-Rubin R statistics were used to assess convergence and they were found to be around or close to 1, suggesting that stationarity had been reached within the simulated Markov chains for the model. The Bayesian deviance estimates were subsequently obtained for each model specification and the results are summarized in Table 7. Among the six model specifications considered, the ones with half-*t* and half-Cauchy prior densities had the smallest *DIC* and/or expected posterior deviance ($\bar{D}$) values. Therefore, the 3PNO model with a half-*t* or half-Cauchy hyperprior provided the best description of the data compared with other model specifications, even after penalizing for a large effective number of parameters (e.g., *p*_*D*_ = 987.24 for the half-*t*, and *p*_*D*_ = 993.80 for the half-Cauchy). On the other hand, with slightly larger deviances, the model with an informative inverse-gamma (3, 2) hyperprior described the data less adequately than those with other prior specifications. The relatively small differences in deviances suggested the actual variability might be close to 0. Furthermore, the model with a half-normal or half-*t* hyperprior had a larger *p*_*D*_ than that with a non-informative prior density. Given the findings from the second simulation study in Section 4, this indicated that the actual standard deviation for the item slope and/or intercept parameters with the data was not larger than 1.

**Table 7:** **Bayesian deviance estimates for the six prior specifications with the ***CBASE*** data**.

***prior*_*αβ*_**	**$\bar{D}$**	**$D\left(\bar{\vartheta }\right)$**	***p*_*D*_**	***DIC***
1	33639.68	32648.78	990.90	34630.58
2	33638.82	32649.49	989.33	34628.15
3	33696.66	32728.93	967.73	34664.40
4	33627.67	32633.87	993.80	34621.47
5	33646.64	32659.98	986.66	34633.31
6	33632.35	32645.11	987.24	34619.59

## 6. Concluding remarks

The half-*t* family offers a good alternative parametric family for the prior distribution of scale hyperparameters in Bayesian hierarchical modeling. This paper adopts it as the hyperprior for item scale parameters in the hierarchical 3PNO IRT model, with the focus being on the weakly informative half-*t* distributions. Their utility in parameter estimation and model comparison has been explored and the results show that they do offer advantages over the commonly adopted uniform or inverse-gamma prior density by allowing the variability for item slope and/or intercept parameters to be either very small or large. The weakly informative half-*t*, and especially the weakly informative half-Cauchy density provides certain level of prior information while it still allows occasional large values. Hence, it overcomes problems resulting from using either a non-informative or an informative prior density when prior information is desired but not readily available. Consequently, the flat-tailed half-*t* distributions are applicable in a wide range of applications and are recommended. In particular, when prior information is not readily available but non-informative priors are not desired, the use of a half-Cauchy distribution is recommended with the scale set to a finite value that is higher than the actual standard deviation. It has to be noted that this paper only focuses on weakly informative half-*t* distributions. One may set the scale of the distribution to be large, e.g., *s* = 100, to make it non-informative. For a non-informative but proper prior distribution, a half-normal with the scale *s* set to a high value, such as 100 should be considered.

The use of the half-*t* prior density for the slope/intercept scale hyperparameter has also an effect on estimating person parameters in 3PNO models. Based on results from the first simulation study, we can see that it tends to result in smaller bias and error in estimating them for data sizes > 10,000. For small data sizes, the use of half-normal prior for item scale hyperparameters may not be suggested over the uniform or inverse-gamma (0.001, 0.001) prior if the focus is primarily on estimating person ability parameters. One may need to further explore the use of other half-*t* prior densities, such as the half-Cauchy, under these conditions. It would also be interesting to find out the reason why manipulating the hyperpriors for item parameters has such an effect on estimating person parameters in the hierarchical 3PNO model, and/or investigate the effect of different (hyper)priors for person parameters in estimating the model.

Further, results and hence conclusion of the second simulation study are based on tests with *k* = 20 and *n* = 1000. Although it is believed that similar results can be obtained with other test lengths (e.g., *k* = 10 or *k* > 20) and/or larger sample sizes (*n* > 1000), additional studies are needed to confirm this and to further investigate the use of half-Cauchy or other half-*t* prior densities for item scale hyperparameters in smaller sample size conditions (e.g., *n* < 500).

In this study, due to the computational expense of MCMC procedures, only 25 replications were adopted and hence the simulation results are based on a limited number of replications. Future studies shall follow the procedure illustrated by Koehler et al. (2009) to ensure the adequacy of the number of replications. Alternatively, one may consider using variational Bayes as suggested by Natesan et al. (2016) given its improved computational efficiency and equivalent estimation accuracy when compared with MCMC. In addition, only certain prior or hyperprior densities for item slope and intercept scale hyperparameters were investigated in this paper. Future research may include more prior specifications or adopt non-conjugate priors for them. Finally, in this study, only Bayesian deviance was used to evaluate individual models. Given that DIC may be limited in that it is not invariant to parameterization and sometimes can produce unrealistic results, further studies can adopt other methods for model comparisons, such as Bayes factors (Kass and Raftery, 1995) or posterior predictive model checking (Rubin, 1984).

## Author contributions

YS designed the study, carried out all the analyses and wrote the manuscript.

### Conflict of interest statement

The author declares that the research was conducted in the absence of any commercial or financial relationships that could be construed as a potential conflict of interest.
